# Role of Pectinolytic Enzymes Identified in *Clostridium thermocellum* Cellulosome

**DOI:** 10.1371/journal.pone.0116787

**Published:** 2015-02-06

**Authors:** Soumyadeep Chakraborty, Vania O. Fernandes, Fernando M. V. Dias, Jose A. M. Prates, Luis M. A. Ferreira, Carlos M. G. A. Fontes, Arun Goyal, Maria S. J. Centeno

**Affiliations:** 1 Department of Biotechnology, Indian Institute of Technology Guwahati, Guwahati, Assam, India; 2 CIISA-Faculdade de Medicina Veterinária, Universidade de Lisboa, Lisboa, Portugal; Universidade Nova de Lisboa, PORTUGAL

## Abstract

The cloning, expression and characterization of three cellulosomal pectinolytic enzymes viz., two variants of PL1 (PL1A and PL1B) and PL9 from *Clostridium thermocellum* was carried out. The comparison of the primary sequences of PL1A, PL1B and PL9 revealed that these proteins displayed considerable sequence similarities with family 1 and 9 polysaccharide lyases, respectively. PL1A, PL1B and PL9 are the putative catalytic domains of protein sequence ABN54148.1 and ABN53381.1 respectively. These two protein sequences also contain putative carbohydrate binding module (CBM) and type-I dockerin. The associated putative CBM of PL1A showed strong homology with family 6 CBMs while those of PL1B and PL9 showed homology with family 35 CBMs. Recombinant derivatives of these three enzymes showed molecular masses of approximately 34 kDa, 40 kDa and 32 kDa for PL1A, PL1B and PL9, respectively. PL1A, PL1B and PL9 displayed high activity toward polygalacturonic acid and pectin (up to 55% methyl-esterified) from citrus fruits. However, PL1B showed relatively higher activity towards 55% and 85% methyl-esterified pectin (citrus). PL1A and PL9 showed higher activity on rhamnogalacturonan than PL1B. Both PL1A and PL9 displayed maximum activity at pH 8.5 with optimum temperature of 50°C and 60°C respectively. PL1B achieved highest activity at pH 9.8, under an optimum temperature of 50°C. PL1A, PL1B and PL9 all produced two or more unsaturated galacturonates from pectic substrates as displayed by TLC analysis confirming that they are endo-pectate lyase belonging to family 1 and 9, respectively. This report reveals that pectinolytic activity displayed by *Clostridium thermocellum* cellulosome is coordinated by a sub-set of at least three multi-modular enzymes.

## Introduction

Plant cell wall degradation carried out by saprophytic and phytopathogenic microbes is essential for the recycling of carbon stored in plant biomass and of intrinsic biotechnological importance. Plant cell walls are composed of a complex network of polysaccharides, primarily cellulose, hemicelluloses and pectic substances [1]. Pectins are a highly heterogeneous group of polymers, containing a high quantity of galacturonic acid, which contribute to the firmness and structure of plant tissues, and is predominantly found in the primary cell wall and middle lamella. This recalcitrant carbohydrate is more soluble in water than cellulose and hemicelluloses, suggesting that it constitutes the initial target for plant associated microbes attack [2]. Pectins are divided into three polysaccharides i.e., homogalacturonan (HG), rhamnogalacturonan type-I (RG-I), and rhamnogalacturonan type-II (RG-II). HG is present as a linear backbone, while RG-I and RG-II are branched carbohydrates [3]. The backbone of RG-I pectin is composed of alternating rhamnose and galacturonic acid residues with a disaccharide repeated unit consisting of [(1,2)-α-L-rhamnose-(1,4)-α-D-galacturonic acid] where galacturonic acid residues may be acetylated at the O2 or O3 positions [4,5]. It is often referred as “hairy” because of the presence of multiple side chains of neutral polymers like arabinans, galactans and arabinogalactans which are attached to C4 of the rhamnose residues [6,7]. RG-II consists of a polygalacturonan backbone with side chains complexes of about 30 monosaccharides including rare molecules such as apiose and aceric acid [8].

Due to its structural complexity, pectin degradation requires the concerted action of several enzymes. The enzymatic degradation of polygalacturonan involves two well-known enzymatic mechanisms: i) hydrolysis by glycoside hydrolases (GH) that cleave glycoside bonds in the polysaccharide and ii) β-elimination reactions carried out by polysaccharide lyases resulting in oligomers with Δ4,5 unsaturated residues at the non-reducing end [9, 10]. Polysaccharide lyases (EC 4.2.2.-) belong to a large group of enzymes defined as carbohydrate-active-enzymes and have been classified into 23 families (May 2014), according to CAZy database [11]. Pectate lyases of families 1, 2, 3, 9, and 10 catalyse the β-eliminative cleavage of α(1→4)-glycosidic bond between D-galactopyranosyluronic acid (Gal*p*A) residue in pectate (a low methylesterified form of pectin), and generate Δ4,5 unsaturated Gal*p*A as the product [12], which exhibits a maximum absorbance at around 235 nm [13].

Pectate lyases are widely distributed among microbial plant pathogens like *Erwinia* [14, 15, 16] although they have also been found in saprophytic bacteria including the genus *Bacillus* [17, 2, 18] and *Clostridium* [12]. *Clostridium thermocellum* is an anaerobic, saccharolytic and thermophilic bacterium that organizes a consortium of plant cell wall degrading enzymes in a large multienzymatic complex termed the cellulosome [19, 20]. The cellulosome is assembled via the interaction of individual type-I dockerins located at the C-terminus of enzymes into one of the nine cohesins of the scaffoldin subunit, CipA. CipA also bears a family 3 carbohydrate-binding module (CBM) which accounts for its cellulose-targeting function and a dockerin type-II that mediates the attachment of the entire complex into the bacterial cell surface [19, 21]. Despite its specialization in the hydrolysis of crystalline cellulose, the cellulosome contains in addition to several cellulases, an extensive group of hemicellulases, which have been extensively characterized [22, 23, 24, 25, 26], and are believed to increase the accessibility of the bacterium into its primary substrate.

The majority of glycoside hydrolases that attack cellulose and hemicelluloses are modular enzymes consisting of catalytic modules appended to non-catalytic carbohydrate-binding modules (CBMs) [10]. Pectinases on the contrary generally have a relatively simple structure lacking CBMs, which is possibly explained by the accessibility of pectins to soluble biocatalysts [27]. CBMs are described to date into 69 families (May 2014), according to CAZy database (http://www.cazy.org/Carbohydrate-Binding-Modules.html) and continue to expand. The present study provides data indicating that *C. thermocellum* cellulosome secretes modular polysaccharides lyases belonging to PL families 1 and 9. The role of this subset of enzymes in the anaerobic conversion of biomass by cellulosomes was investigated.

## Materials and Methods

### Bacterial strains, plasmids and culture conditions

The *Escherichia coli* strains used in this study were NZYStar (NZYTech Ltd.), BL21 (DE3) and BL21(DE3) pLysS (Novagen). The plasmid vectors used were pNZY28 (NZYTech Ltd.), pGEM-T Easy vector (Promega), pET21a and pET28a (Novagen). *E. coli* strains containing recombinant plasmids were cultured in LB broth medium supplemented with 100 μg/mL ampicillin or 50 μg/mL kanamycin. To generate the recombinant proteins encoded by pET21a or pET28a expression vectors, *E. coli* BL21 (DE3) were cultured at 37°C to mid-exponential phase (A_550_ = 0.6) and at this point isopropyl-β-D-thiogalactoside (IPTG) was added to a final concentration of 1 mM. Incubation conditions after induction with IPTG were 16h at 19°C for PL1A and PL9, 12h at 24°C for PL1B.

### Substrates used in enzyme assays

Polygalacturonic acid (PGA) from citrus fruits, rhamnogalacturonan from soyabean (RGAS) and potato (RGAP), pectic galactans from potato (PGP) and lupin (PGL), were purchased from Megazyme. Pectins from citrus fruits (with varying degrees of methyl-esterification, PC) and apple (PA) were purchased from Sigma Chemical Co., USA.

General recombinant DNA procedures

Bacterial transformation, agarose gel electrophoresis, plasmid DNA preparation, restriction endonuclease digestion and ligation of DNA sequences were followed as described elsewhere [28].

### Construction of recombinant plasmids

Genes encoding PL1A, PL1B and PL9 were amplified from *C. thermocellum* genomic DNA, using one IU of thermostable DNA polymerase NZYSpeedy Proof (NZYTech Ltd.) and primer pairs, described in Table 1. *Nhe*I/*Xho*I restriction sites were used for directional cloning of the respective amplified DNA sequences into the expression vectors pET21a and pET28a. The reactions, in a final volume of 50 μl, were subjected to 30 cycles at the following temperatures: 95°C for 1 min, 55°C for 1 min and 72°C for 2.5 min. The amplified genes after running on agarose gel were purified by gel extraction kit (Qiagen), and were cloned into pNZY28 (NZYTech Ltd) or pGEMT-Easy (Promega) and sequenced to ensure that no mutation occurred during PCR. Recombinant pNZY28 and pGEMT-Easy derivatives were digested with *Nhe*I/*Xho*I restriction enzymes (NZYTech Ltd. or Promega). *pL*1a and *pL*9 genes were cloned into similarly digested expression vector pET21a(+) whereas *pL*1b gene was cloned into pET28a(+) vector. Recombinant PL1A and PL9 proteins contained a C-terminal His_6_-tag, whereas PL1B contained an N-terminal His_6_-tag.

**Table 1:** Primers used in PCR of *pl1A, pl1B* and *pl9* genes.

**Protein**	**Vector**	**Primers**
PL1A	pET21a(+)	*5′*- ctc**gctagc**accacttatgcccagacc -*3′*
		*5′*- cac**ctcgag**ggagaatgttcccgggac -*3′*
PL1B	pET28a(+)	*5′*- ctct**gctagc**gcgccaagctttgaactg -*3′*
		*5′*- cgcg**ctcgag**ctgctgagtatttttcgg -*3’*
PL9	pET21a(+)	*5′*- ctc**gctagc**gcaatccctgtggaaggagac -*3′*
		*5′*- cac**ctcgag**tttaaatattgcattgtcaag -*3′*

The nucleotides shown in bold are the restriction enzyme sites, which were used to clone amplified genes into the expression vectors pET21a(+) and pET28a(+).

### Expression and purification of PL1A, PL1B and PL9

PL encoding genes were expressed using *E. coli* BL21 (DE3) where the cells were induced with 1mM IPTG, only when the culture OD *A*_600_ reaches 0.4–0.6. Induced cells were grown overnight at 24°C and 180 rpm for protein production. Cells were harvested by centrifugation at 12000 g, 4°C for 20 min and the bacterial pellets were resuspended in 50 mM sodium HEPES buffer (pH 7.5) containing 1 M NaCl, 5 mM CaCl_2_ and 10 mM imidazole for PL1A and PL9. PL1B containing cells were resuspended in 50 mM Tris-HCl buffer (pH 8.6) containing only 100 mM NaCl. These three recombinant proteins containing His-tags were purified by immobilized nickel ion affinity chromatography as described previously [29]. For PL1A and PL9 the buffer was exchanged to 50 mM Tris-HCl, pH 8.5, containing 5 mM CaCl_2_ and 100 mM NaCl and for PL1B the buffer was exchanged to 50 mM Tris-HCl (pH 8.6) containing 100 mM NaCl. The purity and size of recombinant enzymes were evaluated by SDS-PAGE [30].

### Enzyme assays

The enzyme activity of PL1A, PL1B and PL9 was determined against different pectic substrates. 30 μg of PL1A or PL9 was incubated with 0.5% (w/v) of substrate dissolved in 50 mM Tris-HCl buffer pH 8.5 containing 5 mM CaCl_2_ and 100 mM NaCl at 60°C for 20 min. The assay of PL1B was carried out by incubating 7 μg of enzyme with 0.1% (w/v) of substrate in 50 mM Glycine-NaOH buffer (pH 9.8) containing 0.6 mM CaCl_2_ for 15 min at 50°C. The reactions were stopped by incubation on ice for 10 min and centrifuged at 13,000 *g* for 5 min. The supernatant containing the released unsaturated products was measured by spectrophotometer (Ultrospec III Pharmacia and Cary 100 Bio Varian). The molar extinction coefficient used for the unsaturated product released at A_232_ nm, was 5,200 M^−1^cm^−1^ [31] and at A_235_ nm, was 4,600 M^−1^cm^−1^ [32]. 1 Unit of enzyme was defined as the amount of enzyme that forms 1 μmol of 4,5-unsaturated product per minute, under the described assay conditions.

To determine the maximum activity of PL1A and PL9 at different pH values, all enzymes were incubated with appropriate substrates at 50°C in the following buffers: 50 mM MES (pH 6.5); 50 mM Tris-HCl (pH 7.0 to 8.5) and 50 mM NaHCO_3_ (pH 9.0 to 12.0), and the activity was determined at A_232_ nm as described above. Activity of PL1B at different pH values was determined by incubating with PGA at 50°C using following buffers: 50 mM Tris-HCl (pH 7.6–8.8), 50 mM Glycine-NaOH (pH 9.0–10.6) and 50 mM Na_2_HPO_4_-NaOH (10.8–12), and the activity was determined at A_235_ nm as mentioned earlier. The optimal activities of PL1A and PL9 at a range of temperature from 10 to 100°C, were determined spectrophotometrically at A_232_ nm, by incubating the enzymes in 50 mM Tris-buffer pH 8.5, for 20 min. The optimal activity of PL1B in the temperatures range from 10 to 100°C was spectrophotometrically determined at A_235_ nm in 50 mM Glycine-NaOH buffer pH 9.8, after 15 min of incubation. The thermostability of PL1A and PL9 was evaluated, by incubating the enzyme at different temperatures (30 to 100°C) in 50 mM Tris-HCl pH 8.5, and for PL1B the enzyme was incubated at same temperature range in 50 mM Tris-HCl (pH 8.6) for 30 min [33], and then the residual activity was measured by assay methods described earlier.

Kinetic parameters of these three Clostridial enzymes were measured against PGA (citrus) having an average molecular weight approximately, 25000 g/mol [34]. 20 μl (1.5 mg/ml) of enzyme (PL1A or PL9) was used in 1 ml reaction mixture containing 50 mM Tris-HCl (pH 8.5), 5 mM CaCl_2_ with varying concentrations (0.01 to 0.5% w/v) of PGA were incubated at 50°C. The unsaturated product formation was monitored spectrophotometrically at A_232_ nm. Similarly, 20 μl (1.4 mg/ml) of PL1B was used in 1 mL of reaction volume containing 50 mM Glycine-NaOH (pH 9.8), 0.6 mM CaCl_2_ and varying concentration of PGA (0.01 to 0.5% w/v). The reaction mixture was incubated at 50°C and released unsaturated product was monitored spectophotometrically at A_235_ nm. K_cat_ and K_m_ were determined using the Michaelis-Menten equation. All the reactions were carried out in triplicate and results were reported as mean±SD.

### Analysis of enzyme degradation products

PL1A (6 μg), PL1B (7 μg) and PL9 (6 μg) were separately incubated in 1 ml reaction volume containing 0.1% (w/v) PGA or citrus pectin (25% methyl-esterified). The reaction was carried out under optimized conditions of pH and temperature for different time intervals from 0 to 60 min. After the reaction enzyme was deactivated by keeping on ice for 5 min and the sample was treated with equal volumes of ethanol to precipitate un-hydrolyzed polysaccharides and protein. Ethanol was removed and samples were concentrated to 500 μl by heating at 50°C. 1 μl of sample was then loaded on the TLC plate (readymade silica coated aluminum TLC plates obtained from Merck, Germany) for running the degradation products under a solvent system containing butan-1-ol/water/acetic acid in the ratio of 5:3:2 [35]. The spots on TLC plates were visualized by a solution containing 0.5% (w/v) α-naphthol and 5% (v/v) sulphuric acid in ethanol [36], after heating at 95°C for 10 min in hot air oven. Standard oligogalacturonides like D-galacturonic acid (S1), di-galacturonic acid (S2) and tri-galacturonic acid (S3) (procured from Sigma Chem. Co., USA) were used to analyze the degradation product formed from different substrates upon enzymatic treatment.

## Results

### Molecular architecture of three pectin degrading enzymes from *Clostridium thermocellum*

**Figure 1:**
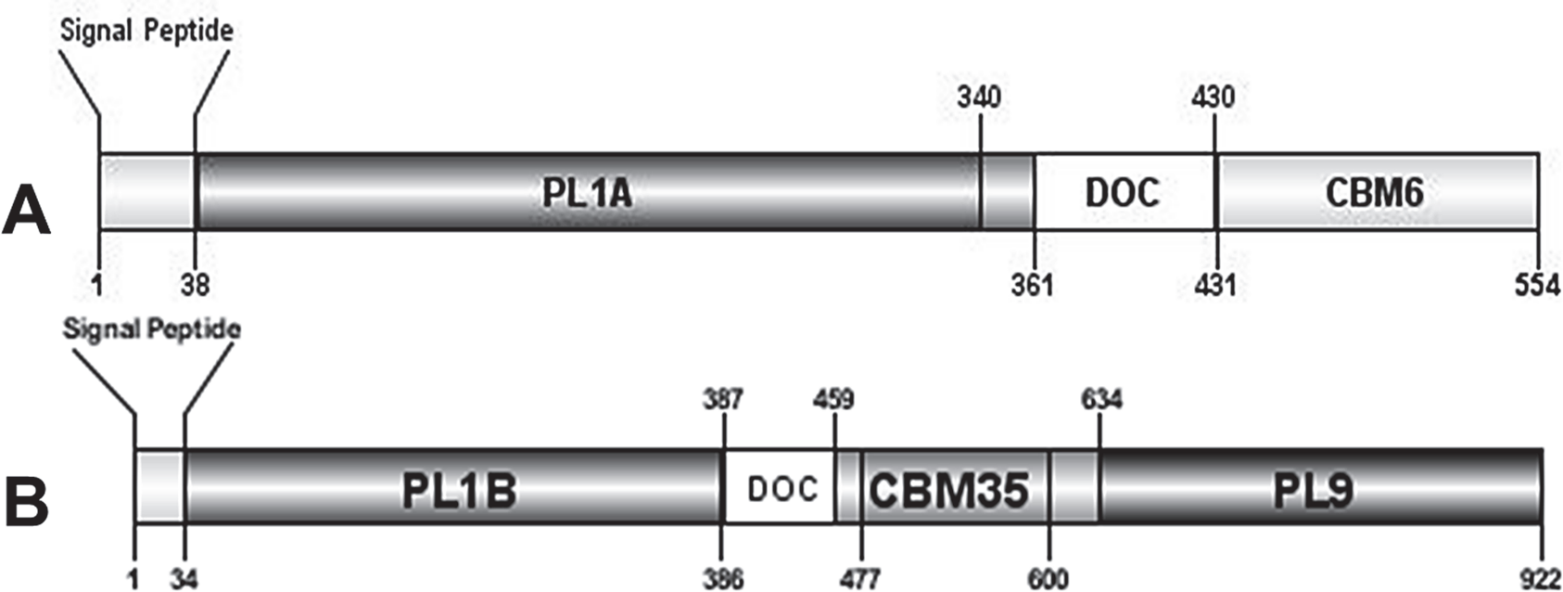
Molecular architecture of modular (A) protein sequence with accession no. ABN54148.1 that comprises of N-terminal PL1A catalytic domain, followed by DOC, type-I dockerin and C terminal CBM6 binding domain. (B) protein sequence with ac no. ABN53381.1 that comprises of N terminal PL1B catalytic domain, followed by DOC, type-I dockerin and CBM35 binding domain, with a C-terminal PL9 catalytic domain.

**Figure 2:**
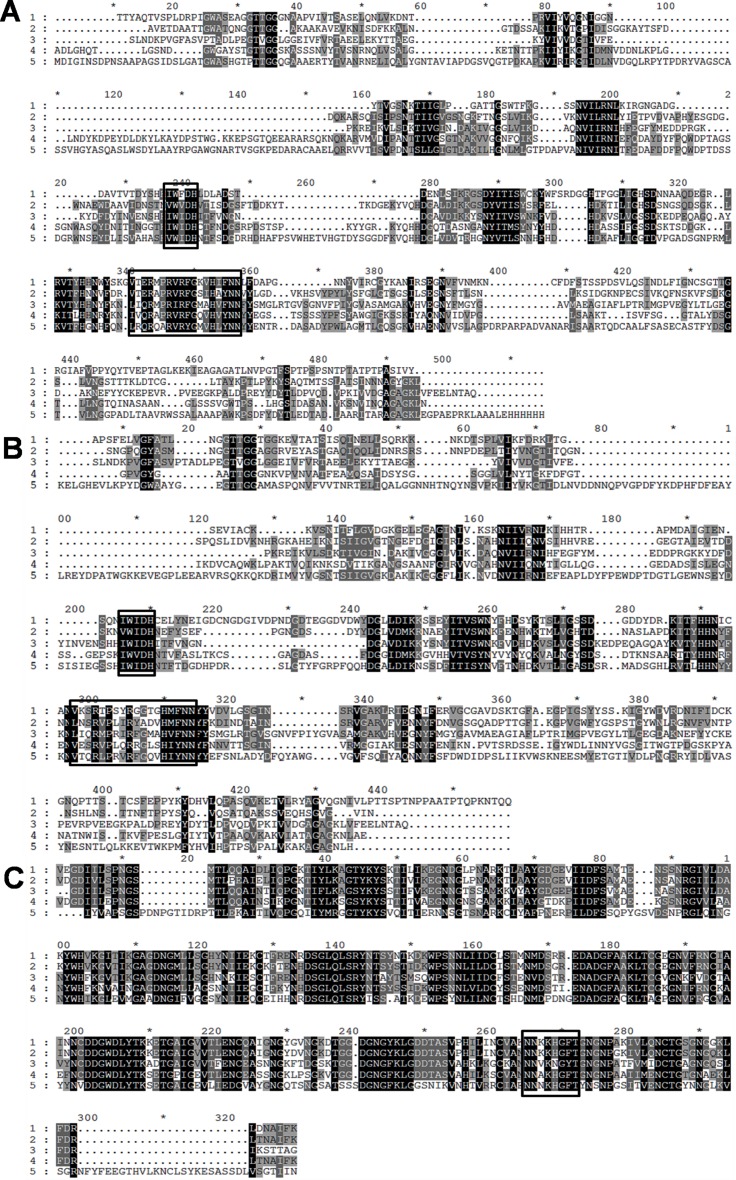
Multiple Sequence Alignment of family 1 and 9 polysaccharide lyases performed by CLUSTALW program and viewed in GeneDoc ver2.7. Identical amino acid residues common in all sequences are represented by black shade, and similar amino acids which are common in atleast 3 out off 5 sequences are represented by grey shades. Consensus sequences that characterize pectate lyases are inside of black squares. (A) PL1A alignment was with following proteins: 1 (PL1A, *Cl. thermocellum ATCC 27405*); 2 (*Erwina chrysanthemi*, PDB: 1PCL); 3 (*Thermotoga maritima*, PDB: 3ZSC); 4 (*Bacillus subtilis*, PDB: 3KRG) and 5 (*Acidovorax Avenae Subsp Citrulli*, PDB: 4HWV). (B) PL1B was aligned with following proteins: 1 (PL1B, *Clostridium thermocellum ATCC 27405*); 2 (*Bacillus Sp*. N16–5, PDB: 3VMV); 3 (*Thermotoga Maritima*, PDB: 3ZSC); 4 (*Xanthomonas Campestris* ATCC 33913, PDB: 2QX3); 5 (*Bacillus Sp*. TS-47, PDB: 1VBL). (C) PL9 was aligned with following proteins: 1 (PL9, *Clostridium thermocellum ATCC 27405*); 2 (*Clostridium straminisolvens* JCM 21531, GAE89695.1); 3 (Clostridium cellulovorans 743B, YP_003842407.1); 4 (*Acetivibrio cellulolyticus*, WP_010245176.1); 5 (*Caldicellulosiruptor kristjanssonii* I77R1B, YP_004026944.1).

Inspection of two protein sequences from *C. thermocellum* revealed that they contain one gene (*pL*1a) in the sequence ABN53381.1 (Fig. 1A) and two genes (*pL*1b and *pL*9) in the sequence ABN53381.1 (Fig. 1B) all putatively expressing lyase activities. Both the protein sequences are associated with type I dockerin, which is the signature module of the cellulosomal proteins. Analysis of the deduced amino acid sequence of the three enzymes revealed characteristic N-terminal signal sequences with putative cleavage sites located between Ala-37/Thr-38 (ABN54148.1) and between Ala-33/Ala-34 (ABN53381.1) suggesting that the proteins are exported into the extracellular space. Homology searches using Blast (www.ncbi.nlm.nih.gov/BLAST), revealed downstream the signal peptide of ABN54148.1 a putative 302-aa family 1 PL (PL1A) followed by a 70-aa dockerin domain and a 124-aa C-terminal family 6 CBM (CBM6) (Fig. 1A). ABN53381.1 contains a 353-aa N-terminal family 1 PL (PL1B) and a 289-aa C-terminal family 9 PL (PL9). Sandwiched between these two catalytic domains is a 73-aa dockerin domain and 124-aa family 35 CBM (CBM35) (Fig. 1B). Alignment of PL1A (Fig. 2A) and PL1B (Fig. 2B) domain with other PL1 homologues revealed two consensus sequence patterns in the enzymes catalytic domains, “VWIDH” and “VxxRxPxxRxGxxHxxxN”, which are signature regions of pectate lyases (Pel) [37, 38, 39]. The conserved arginine residue observed in the second region, identified as R-218 in the superfamily pectate lyase C (PelC from *Erwinia chysanthemi*) is the catalytic residue involved in proton abstraction [40, 41]. Cleavage of glycosidic bonds in which the aglycone sugar is galacturonic acid can be acid-base-assisted catalysis, mediated by glycoside hydrolases [42] or via β-elimination reaction, which is initiated by proton abstraction from C-5 of the galacturonosyl residue on the reducing end of the glycosidic bond [43]. As R-218 belongs to a potential group or groups involved in the proton abstraction in PelC and as it is highly conserved in catalytic domains of PL1A (position 190 in Fig. 2A) and PL1B (position 209 in Fig. 2B) it suggests that these three enzymes cleave α-1,4-linked galacturonic acid units of the pectate component of the plant cell wall by a β-elimination mechanism as expected for pectate lyases. The “VWIDH” region is highly conserved in PL1A and PL1B (Fig. 2A and 2B) and it is involved in the membrane transport and in the protein fold [44]. Structural motifs (parallel β-helix) are also identified in pectate lyases from *Erwinia chysanthemi* and *Bacillus subtilis* [40, 45, 46].

PL9 catalytic domain bears profound similarity with Pel9A from *Erwinia chryanthemi*. Pel9A showed an endolytic cleavage pattern where it cleaves the polysaccharide by anti-β-elimination mechanism, where a base catalyzed abstraction of proton is carried out from the C5 carbon [47]. In case of Pel9A the putative base is Lysine rather than Arginine found in other endo-pectate lyases [48]. Such a consensus sequence was found in the catalytic domain of PL9, whose translated amino acid sequence is highly conserved in all the aligned sequences marked within a box in Fig. 2C. The lysine residue involved in proton abstraction during β-elimination is located in position 269 of the PL9 sequence (Fig. 2C).

### Cloning, expression and purification of recombinant PL1A, PL1B and PL9

**Figure 3:**
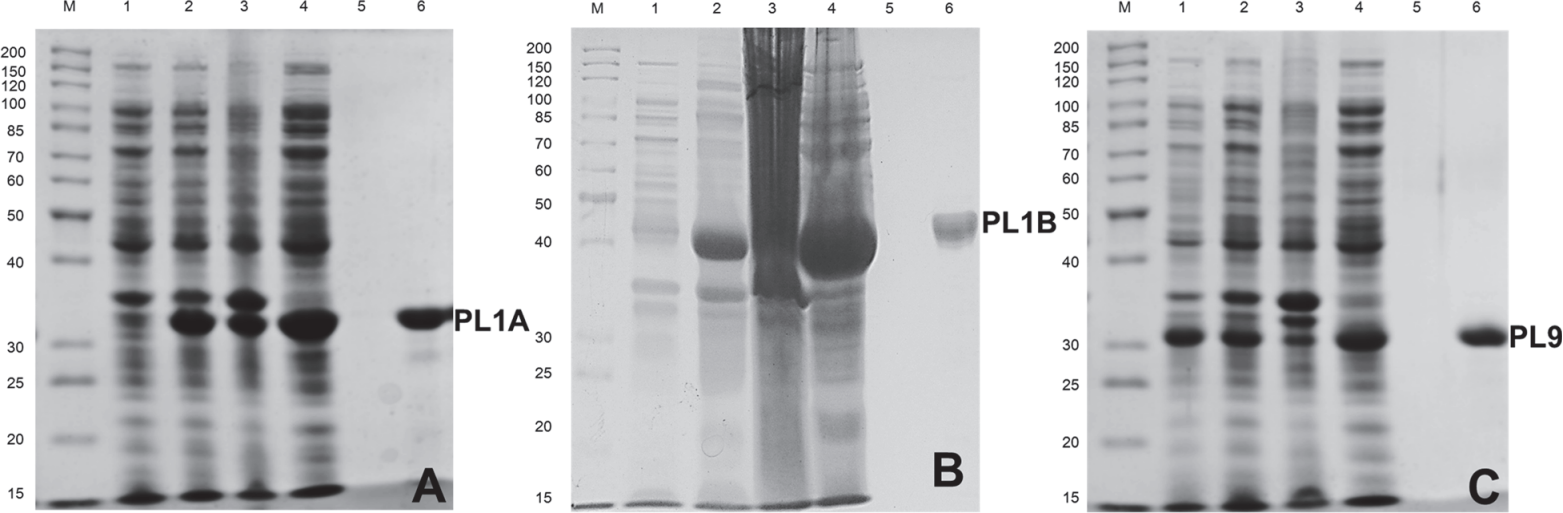
Hyper-expression and purification of PL1A, PL1B and PL9 using *E. coli* BL21 (DE3) cells. The purity of the proteins was analysed by SDS-PAGE using 10% (w/v) gel showing **(A)** PL1A (34 kDa); **(B)** PL1B (40 kDa); **(C)** PL9 (32 kDa); Lane M: Fermentas high range protein molecular weight marker; Lane 1: Uninduced BL21 cells; Lane 2: IPTG induced BL21 cells; Lane 3: Cell pellet after sonication; Lane 4: Cell free extract, Lane 5: Last wash from column and Lane 6: Purified recombinat enzyme.

DNA sequences of 906, 1059 and 867 bp, encoding PL1A, PL1B and PL9 respectively, were amplified by PCR and cloned into pET21a and pET28a expression vector as described in the method section. The recombinant proteins containing the His_6_-tags were purified by immobilized metal ion affinity chomatography. The expression and purification of PL1A, PL1B and PL9 proteins was analyzed by SDS-PAGE as shown in Fig. 3A, B and C displaying molecular size of approximately 34, 40 and 32 kDa, respectively.

### Biochemical properties of PL1A, PL1B and PL9

**Figure 4:**
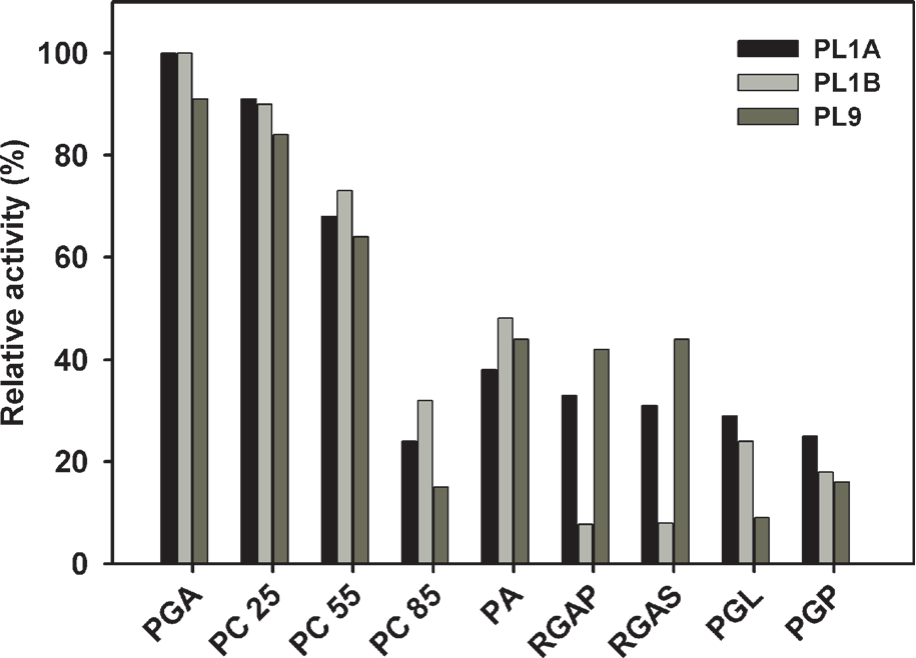
Substrate specificity of PL1A, PL1B and PL9 towards pectic polysaccharides, where PGA: Polygalacturonic acid, PC 25: Pectin (Citrus) (25% methyl-esterified), PC 55: Pectin (Citrus) (55% methyl-esterified), PC 85: Pectin (Citrus) (85% methyl-esterified), PA: Pectin (Apple), RGAP: Rahmnogalacturonan (Potato), RGAS: Rahmnogalacturonan (Soyabean), PGL: Pectic galactan (Lupin), PGP: Pectic galactan (Potato).

The biochemical role of *C. thermocellum* cellulosomal PL1A, PL1B and PL9 enzymes was investigated by analyzing their activity against different substrates. All the three enzymes PL1A, PL1B and PL9 were predominantly active towards polygalacturonic acid (PGA) and pectin both from citrus (Fig. 4). PL1B displayed relatively higher activity with 55% and 85% methyl-esterified pectins from citrus than PL1A and PL9. On the other hand PL1A and PL9 showed 30–40% relative activity with rhamnogalacturonan from potato (RGAP) and soyabean (RGAS), whereas PL1B showed only 8% relative activity.

**Figure 5:**
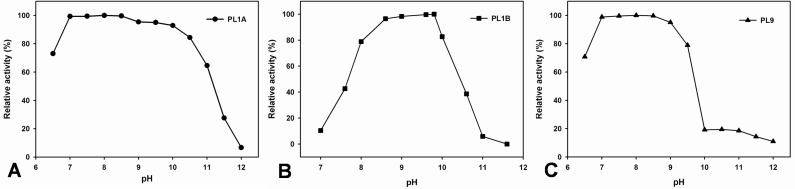
Effect of pH on the activity of (A) PL1A; (B) PL1B; (C) PL9 towards PGA as substrate.

The effect of pH and the temperature on the activity of the recombinant PL1A, PL1B and PL9 enzymes against PGA was determined. The results showed that PL1A, PL1B and PL9 were active under alkaline conditions. PL1A and PL9 were active within pH range (6.5–9.5) showing highest activity at pH 8.5 (Fig. 5A and C). PL1B was active within pH range (8–10) displaying highest activity at pH 9.8 (Fig. 5B).

**Figure 6:**
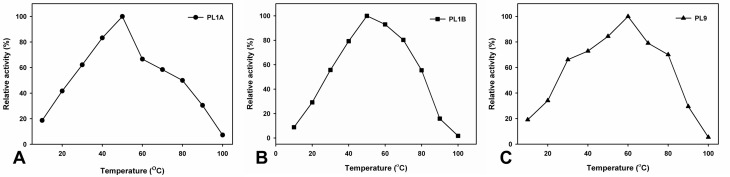
Effect of temperature on the activity of (A) PL1A; (B) PL1B; (C) PL9 towards PGA as substrate.

**Figure 7:**
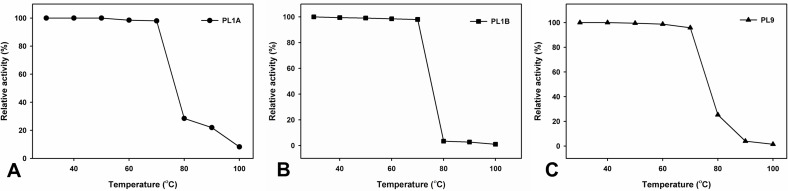
Thermostability of (A) PL1A; (B) PL1B; (C) PL9 towards PGA, after 30 min of incubation of the enzyme at different temperatures.

The optimum temperature was found at 50°C for both PL1A and PL1B and 60°C for PL9 (Fig. 6A, B and C) which were expected because these enzymes originated from a thermophilic bacterium. Nevertheless all the three recombinant enzymes, PL1A, PL1B and PL9 displayed thermostability within the temperature range of 30 to 70°C for 30 min (Fig. 7A, B and C).

**Figure 8:**
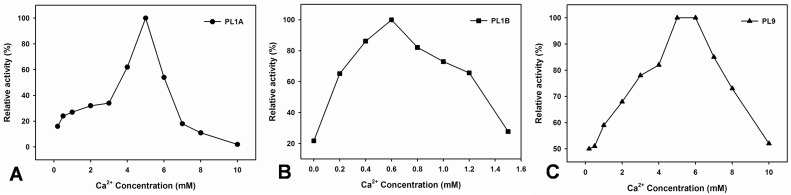
Effect of concentration of Ca^2+^ ions on the activity of (A) PL1A; (B) PL1B; (C) PL9 against PGA as substrate.

All the three enzymes showed an exclusive requirement of Ca^2+^ ions to achieve their maximum activity. PL1A and PL1B showed only 20% of their maximum activity in the absence of 5 mM and 0.6 mM Ca^2+^ ions, respectively, whereas PL9 showed 50% of its maximum activity in absence of 5 mM Ca^2+^ ions (Fig. 8A, B and C). The optimum Ca^2+^ ion concentration required to achieve 100% pectinolytic relative activity were 5 mM for both PL1A and PL9, whereas 0.6 mM for PL1B.

Kinetic parameters of these three enzymes were determined against PGA and are presented in Table 2. The data revealed that PL1A, PL1B and PL9, showed turnover number values (K_cat_) of 1.3, 1.76, and 1.32 min^−1^ respectively (Table 2). The catalytic efficiency (K_cat_/K_m_) values exhibited by PL1A, PL1B and PL9 were 41, 62 and 35 mM^−1^min^−1^ respectively, revealing that PL1B exhibit higher catalytic efficiency on PGA, than PL1A and PL9.

**Table 2:** Kinetic parameters of PL1A, PL1B and PL9 with Polygalacturonic Acid (PGA) from citrus.

**Enzyme**	**Substrate**	**K_cat_ (min^−1^)**	**K_m_ (mM)**	**K_cat_/K_m_ (mM^−1^ min^−1^)**
**PL1A**	**PGA** (citrus)	1.3±0.03	0.0313±0.0005	41±0.23
**PL1B**	**PGA** (citrus)	1.76±0.05	0.0286±0.0002	62±0.43
**PL9**	**PGA** (citrus)	1.32±0.05	0.0378±0.0004	35±0.4

One unit of enzymatic activity (U) was defined as the amount of enzyme in mg that produces 1 mmol/L of unsaturated product per minute.

### Functional properties of recombinant PL1A, PL1B and PL9

**Figure 9:**
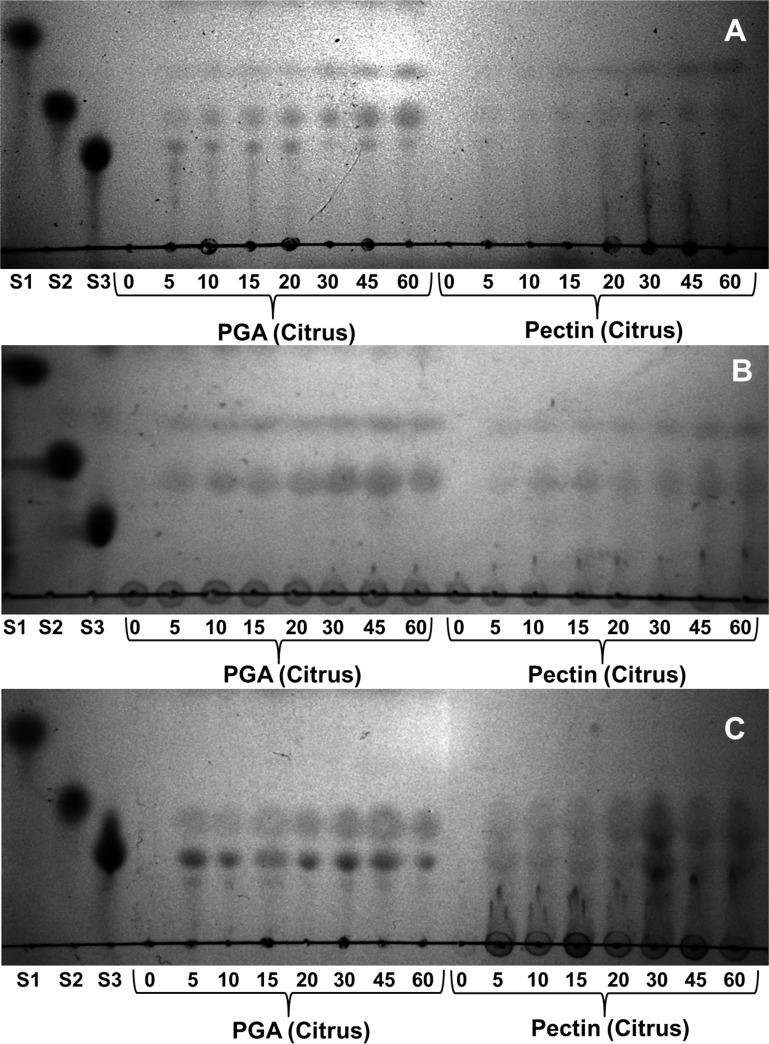
Thin layer chomatography (TLC) showing the enzymatic degradation products of PGA (citrus) and pectin (citrus) (25% methyl-esterified). Chromatogram displaying hydrolysis by (A) PL1A (B) PL1B and (C) PL9 at 0, 5, 10, 15, 20, 30, 45 and 60 min. Standard oligosaccharides used were S1: D-galacturonic acid; S2: Di-galacturonic acid; S3: Tri-galacturonic acid.

**Figure 10:**
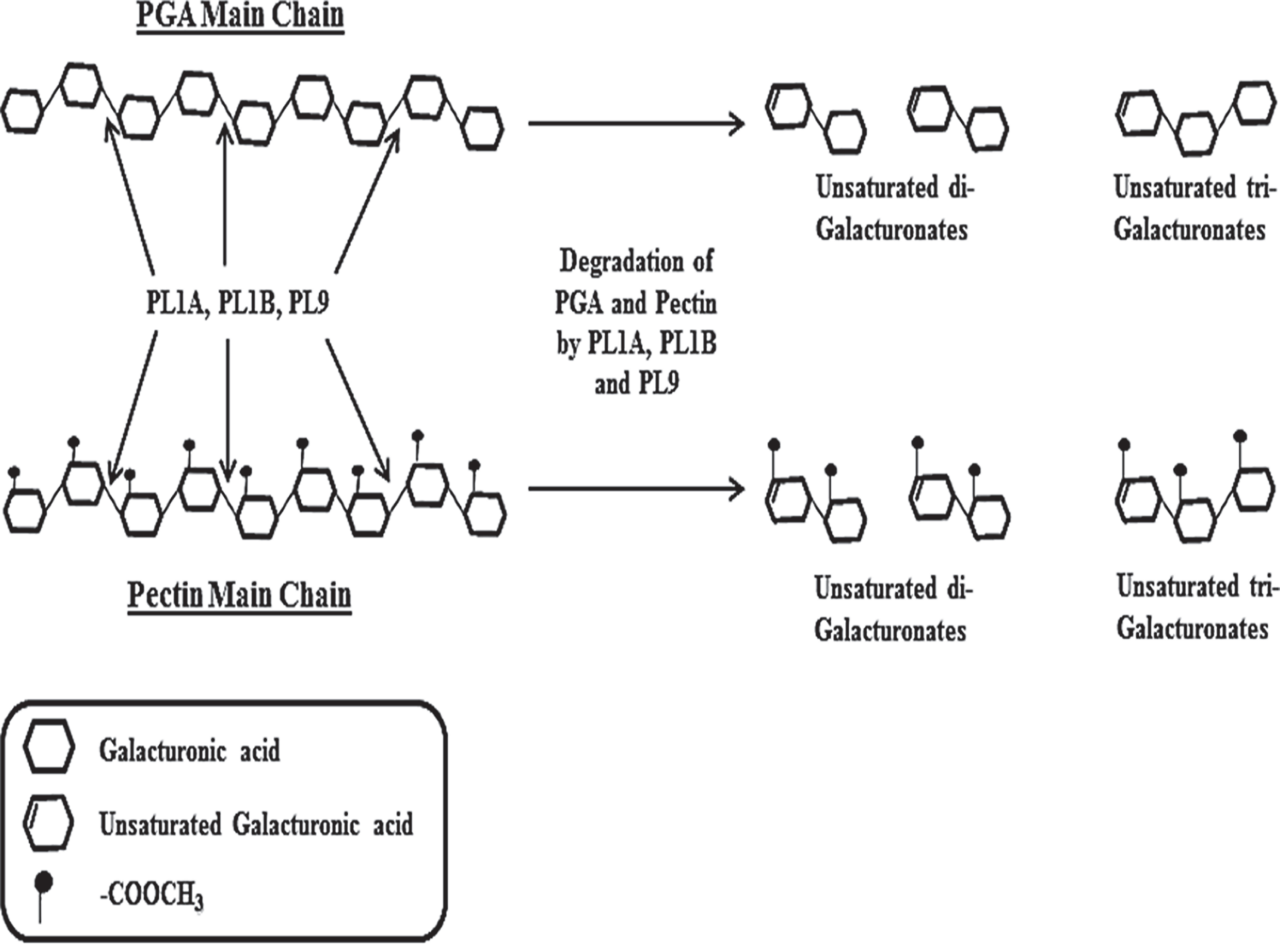
Schematic presentation of mode of action of PL1A, PL1B and PL9 against PGA and pectin (citrus) hydrolysis leading to production of corresponding unsaturated oligo-galacturonates.

Products released by the enzymatic cleavage of PL1A, PL1B and PL9 of PGA and pectin (citrus) were determined. The reactions were carried out under optimum conditions of pH and temperature for each individual enzyme as mentioned in Methods section. The samples from enzymatic reaction were collected at different time intervals of 0, 5, 10, 15, 20, 30, 45 and 60 min and separated through TLC. PL1A produced unsaturated di- and tri-galacturonates along with other oligosaccharides of higher size. The accumulation of unsaturated tri-galacturonates and higher size oligosaccharides was predominant after 5 min of the start of reaction (Fig. 9A). It was evident from the TLC results that an increase in the amount of unsaturated di- and tri-galacturonates was found with increase in time and found to be highest at 60 min of reaction time (Fig. 9A). PL1B from the beginning of the reaction started producing unsaturated di and tri-galacturonates, and no higher size oligosaccharides were observed. Accumulation of unsaturated di- and tri-galacturonates increased with time and found to be highest at 60 min of the reaction (Fig. 9B). PL9 produced unsaturated tri-galacturonates and oligosaccharides of higher size, and the accumulation of this two products were found to be constant from 5 to 60 min of the reaction time (Fig. 9C). The cleavage pattern of these three enzymes suggests that they cleave within the poly-galacturonan main chain of PGA and pectin (citrus) thus following an endo cleaving pattern. The mechanism by which these three enzymes from *Clostridium thermocellum* cleaves the α-1,4 linkages in pectic polysaccharides thus resulting in enzymatic degradation is explained schematically in Fig. 10.

## Discussion

*C. thermocellum* is known to hydrolyze not only cellulose but also hemicelluloses [22, 23, 24, 25, 26, 49]. It was also shown that *C. thermocellum* could utilize polygalacturonic acid and pectins as carbon sources [50]. The results described in this paper demonstrate that *C. thermocellum* cellulosome is composed of enzymes that are able to attack pectin and can hydrolyze these complex polysaccharides. We have identified and characterized for the first time three cellulosomal pectinolytic enzymes PL1A, PL1B and PL9 from this microorganism. The data revealed PL1A, PL1B and PL9 catalytic activity on polygalacturonic acid (PGA) and pectin (citrus). Sequence similarity studies with proteins in biological databanks placed PL1A, PL1B and PL9 in families 1 and 9 of pectate lyases (PL), respectively. Till date there are 22 families of polysaccharide lyases, of which PL families 1, 2, 3, 9 and 10 contain pectate lyases (http://www.cazy.org/search?page=recherche&recherche=4.2.2.2&tag=9).

Analysis of primary sequences of the cellulosomal enzymes under analysis here revealed a modular organization with the presence of a CBM, which is rare in pectinases [12]. It is generally believed that pectins are more accessible to enzyme attack than cellulose and hemicelluloses, and as such it has been assumed that there has been less evolutionary pressure for pectinases to contain CBMs. However, Rgl11A and Pel10A from *P. cellulosa* and Pel4A from *Clostridium cellulovorans* are examples of prokaryotic pectinases that contain a cellulose-binding domain [12, 27, 51]. This report showed that CBMs are prevalent within cellulosomal pectinases and might be involved in potentiating the degradation of less recalcitrant substrates. Previous studies showed that functionally active family 6 and 35 CBM’s bind strongly to cellulose [52, 53]. Family 6 CBMs display considerable promiscuity in ligand binding with different modules showing affinity for amorphous cellulose, xylans and β-glucans [54]. In addition, CBM family 35 also reveals considerable plasticity in ligand recognition which is not surprising considering that this family shares sequence similarities with CBM 6. Both these families, CBM 6 and CBM 35, are structurally related to the β-jelly-roll CBM superfamily [55] and can be viewed as a subfamily of the large β-jelly-roll CBM superfamily [56]. CBMs are prevalent in plant cell wall degrading enzymes and as a general function promote the interaction of the enzyme with their target substrate [55]. PL1A contains a CBM 6, while PL1B and PL9 exhibit a CBM 35. The presence of CBMs in the structure of PL1A, PL1B and PL9 suggests that they are important in increasing their catalytic efficiency by bringing the enzymes into close proximity to their target substrates. However, in a recent work Montanier and colleagues [57] while analysing the biological role of 4 members of family CBM35, it was revealed that the biological role of CBM35s is not dictated solely by the substrate specificity of their appended catalytic domains as members of these CBM family may recognize the products of pectin hydrolysis. Structurally, PL1A, PL1B and PL9 consist of an individual dockerin-containing enzyme integrated into the *C. thermocellum* cellulosome by CipA cohesin-dockerin interaction with a non-catalytic module CBM-like.

PL1A, PL1B and PL9 are characteristic pectate lyases and preferentially hydrolyze polygalacturonic acid, though they also act on pectins. Moreover, all the three enzymes displayed significantly higher activity with 55% and 85% methyl-esterified pectin (citrus). Similar high activity of pectate lyase on pectins with high degree of methyl-esterification has been previously reported only from *Bacillus subtillis* [17]. PL1A and PL9 showed significant activity with rhamnogalacturonan from potato (RGAP) and soyabean (RGAS), as compared with PL1B. The enzymes that degrade the backbones of pectic substances utilize two distinct cleavage mechanisms, the hydrolysis or the β-elimination. The method used to evaluate the PL1A, PL1B and PL9 activities provide evidence that these enzymes are lyases, as they catalyse the β-eliminative cleavage of glycosidic bonds with the production of Δ4,5 unsaturated galacturonates, which can be followed spectrophotometrically at absorbances of 232 to 235 nm. PL1A and PL9 was active within the pH range of 6.5–9.5 with highest activity at pH 8.5, which is similar to those of Rgl11Y from *C. cellulolyticum* cellulosome (pH 8.5) and from *C. cellulovorans* cellulosome Pel4A (pH 8.0) [12, 58], but PL1B was active within the pH range of 8–10 and showed highest activity at pH 9.8 similar to PelA from *Bacillus sp*. which showed pH optima of 10 [59]. All four enzymes have a requirement of Ca^2+^ ions to achieve their maximum activity. It has been suggested that the pH value of some plant tissues changes during microbial attack which possibly indicate that the degradation of plant cell wall polysaccharides occurs sequentially according to the pH of plant tissues [12]. It is also known that pectate lyases require Ca^2+^ for *in vitro* activity and presumably utilize the abundant Ca^2+^ in the plant cell wall for *in vivo* activity [38, 60].

Analysis of the degradation products of cellulosomal PLs by TLC conclusively inferred that PL1A, PL1B and PL9 followed an endo cleavage pattern on PGA and pectin (citrus), cleaving these substrates endolytically as was previously reported for PelA from *Clostridium cellulovorans* [58]. These enzymes produced unsaturated di, tri and higher oligogalacturonates from PGA and pectin (citrus). PelC from *B. subtilis* [17], also an endo pectate lyase, showed a similar cleavage pattern producing mixtures of different degradation products, whereas PelX from *Erwinia chysanthemi* an exo-pectate lyase always produced a single degradation product either unsaturated di or tri-galacturonates [61]. Hence, PL1A, PL1B and PL9 under investigation are conclusively endo pectate lyases. Pectic substrates are highly heterogeneous which may require many enzymes with different specificities and catalytic mechanisms for their complete breakdown. Therefore, the ability of these cellulosomal enzymes to degrade pectic substances suggests that cellulosomes are designed for the degradation of an entire set of carbohydrates within plant cell walls, and not only cellulose and hemicellulose. It is clear that within cellulosomes other enzymes presently of unknown function may target the degradation of pectic polysaccharides.

### Conclusion

Thermostable enzymes are important resources in various industrial processes that occur at higher temperatures. Hence enzymes described in this study will be competent enough for industrial processes like fruit juice extraction, vegetable and fruit maceration or bioscouring of cotton fabric to increase the efficiency of dying at improved temperatures. These enzymes can be used as a cocktail for further efficient and complete degradation of pectic polysaccharides.
